# The Antimicrobial Activity of Essential Oil from *Perovskia abrotanoides* Karel and its Main Components

**DOI:** 10.4103/0250-474X.56016

**Published:** 2009

**Authors:** M. Mahboubi, N. Kazempour

**Affiliations:** Department of Microbiology, Barij Essence Pharmaceutical Company, 87135-1187 Kashan, Iran

**Keywords:** α-pinene, camphor, 1, 8-cineole, *Perovskia abrotanoides*, antimicrobial activity

## Abstract

In Iranian folk medicine, *Perovskia abrotanoides* is used for treatment of leishmaniasis. These patients may develop secondary infections with opportunistic microorganisms. Therefore, the antimicrobial activity of essential oil from aerial part of *P. abrotanoides* and its main components was evaluated against different microorganisms. Disc diffusion and broth micro dilution assays were used for *in vitro* antimicrobial screening. The antibacterial activity of this oil and main components on viability of *S. aureus* was determined. The oil showed antimicrobial activity against *Candida albicans* and Gram positive bacteria especially *Staphylococcus aureus* with zone inhibitions and minimal inhibitory concentration values in the range of 7.6 to 29 mm and 2 to 8 μl/ml respectively, whereas the least susceptible were *Aspergillus niger* and Gram negative bacteria. In viability test, the results showed that the antimicrobial activity of 1,8-cineole was more than that of α-pinene and camphor but after 60 min this effect gradually decreased only for 1,8-cineole and ultimately the antibacterial activity of camphor was more than that of α-pinene. 1,8-cineole had weak antimicrobial activity against all of the tested microorganisms. Hence the use of *P. abrotanoides* oil could be useful in fighting secondary infections in leishmaniasis especially against *S. aureus*.

*Perovskia* genus, Labiatae family has seven species out of which three species such as *P. abrotanoides*, *P. atriplicifolia* and *P. artemisoides* grow in Iran[1]. *P. abrotanoides* with vernacular name of *Brazambal*, *Domou*, and *Gevereh* is a perennial herb growing wild in Iran, Afghanistan, Pakistan and Turkmenistan[2]. People customarily has been used the grinded roots of plant in water, sesame oil and wax and is used like a paste for treatment of leishmaniasis with any scientific base. Attempts have been made to explain the efficacy of this plant for treatment of leishmaniasis[2,3]. Some of the pharmacological effects of plant such as leishmanicidal, antiplasmodial and cytotoxic activity[2,3] as well as antinociceptive and antiinflammatory have been confirmed[4]. The lesions of leishmaniasis become susceptible to colonization or infection with a number of pathogenic or opportunistic fungi and bacteria that cause secondary infections. Lesion care and management of secondary infections are essential in recovery of leishmaniasis[5]. Perhaps the antimicrobial activity of *P. abrotanoides* could contribute to treat leishmaniasis. There was only one report about antifungal activity of essential oil from flowers of *P. abrotanoides* against *Aspergillus niger*, *Aspergillus flavus*, *C. albicans* and *Trichopyhton mentagrophytes*[6]. Inouye *et al* had documented that this oil with main constituents of 1,8-cineole and α-pinene showed no activity against *C. albicans* and fungi[6]. In this investigation, we have evaluated the *in vitro* antimicrobial activity of aerial part of *P. abrotanoides* essential oil and its major components against some microorganisms including Gram positive, Gram negative bacteria, yeast and fungi.

Essential oil from aerial part and its major components *P. abrotanoides* of known composition (α-pinene 12%, camphor 23%, 1,8-cineole 22%, β-pinene 3.1% and limonene 1.5%) was obtained from Barij Essence Pharmaceutical Company. Camphor, α-pinene, and 1,8-cineole were purchased from Merck (Darmstadt, Germany), Roth (Carl Roth KG, Karlsruhe, Germany), respectively. *Staphylococcus aureus* ATCC 25923, *Bacillus cereus* ATCC 1247, *Escherichia coli* ATCC 8739, *Pseudomonas aeruginosa* ATCC 9027, *Candida albicans* ATCC 10231 and *Aspergillus niger* ATCC 16404 were used as tested microorganisms.

For screening of antimicrobial activity disc diffusion[7] and micro broth dilution[9] assays were used. In disc diffusion method, the cultured bacteria were suspended in normal saline but spore of fungi and yeast was inserted in RPMI 1640 (Sigma-Aldrich chemie GmbH, Steinheim, Germany) buffered with 0.165 M morpholine propane sulfonic acid (MOPS, Merck KGaA, Darmstadt, Germany)[8]. The turbidity of microorganisms was adjusted to 0.5 McFarland. The inocula were 1×10^8^, 1×10^6^ CFU/ml for bacteria and fungi respectively. Inoculate was swabbed on Muller Hinton Agar (bacteria) and sabouraud dextrose Agar (fungi) by sterile cotton swab. Sterile blank discs impregnated with 3, 5, 10 μl of oil in 10 μl of dimethylsulfoxide (DMSO) were used and put on cultured plates. Disc containing DMSO and antibiotics were used as control. The plates were incubated at 37, 25 aerobically and the zone inhibition diameters was measured in millimeter (mm) after 24 and 48 h for bacteria and fungi respectively. The minimal inhibitory concentrations (MICs) were determined by micro broth dilution assay[9]. A two fold serial dilution of oil and its main components (1,8-cineole, α-pinene, and camphor) were prepared in 10% DMSO (serial dilution= 32-0.125 μl/ml). Muller Hinton broth[9] and RPMI 1640[8] were used as a broth media for bacteria and fungi respectively. After shaking of dilutions, 100 μl of each dilution was inserted in each well and 100 μl of above microbial suspension was diluted (bacteria ca.1×10^6^, fungi ca. 0.5-1×10^5^ CFU/ml) and then was added to each serial dilution and incubated. MICs were defined as the first well with no visible growth and minimal bactericidal or fungicidal concentrations (MBCs or MFCs) were as the first well that no growth on solid media after 24, 48 h for bacteria and fungi, respectively.

For viability assay of oil and main components against *S. aureus*, five flasks containing 5 ml of cell suspension in Cation adjusted Muller Hinton broth (approximately 5×10^6^ CFU/ml) were prepared. *P. abrotanoides* oil and main components was added at the MIC concentration value to each flask except control. One hundred microlitres of control flask was sampled immediately, serially diluted and placed on nutrient agar to determine the cell density. After 30, 60, 120 min at room temperature, aliquots (100 μl) were removed to neutralizing buffer (0.075 M buffer phosphate pH 7.9), serially diluted and CFU/ml were determined for each components and oil and Log CFU/ml was calculated[10].

The results showed that *C. albicans* and Gram positive bacteria especially *S. aureus* were highly susceptible to the oil. The oil showed no zone inhibition against fungi and Gram negative bacteria (Table 1). The oil exhibited the large inhibition zone against *S. aureus* and the zone inhibitions were enhanced with increasing the amount of essential oil (14.4±1.4 to 29±5.8 mm). The effect of oil was larger than vancomycin.

**TABLE 1:** ANTIMICROBIAL ACTIVITY OF *P. ABROTANOIDES* OIL AGAINST DIFFERENT MICROORGANISMS

Strains	Zone Inhibition Diameter (mm)			
	
	3 μl	5 μl	10 μl	Antibiotics
*S. aureus*	14±1.4	19.6±2.00	29±5.8	Vancomycin 17±0.0
*B. cereus*	10.3±0.47	13±1.4	16±0.82	Erythromycin 26±0.0
*E. coli*	0.0±0.0	0.0±0.0	0.0±0.0	Gentamycin 20±0.0
*P. aeruginosa*	0.0±0.0	0.0±0.0	0.0±0.0	Gentamycin 28±0.0
*C. albicans*	7.6±0.94	11.3±0.47	14±0.81	Amphotricin B 16±0.81
*A. niger*	0.0±0.0	0.0±0.0	0.0±0.0	Amphotricin B 14±0.0

mm is millimeters

The oil showed bactericidal effect against *S. aureus* with MIC and MBC values equal 8 μl/ml. The zone inhibition of *S. aureus* was larger than the zone inhibition of *B. cereus* but the MIC of oil for *B. cereus* was lower than MIC value of *S. aureus* (Table 2). C. *albicans* was more sensitive than *A. niger* to the oil and the oil had fungicidal activity against *C. albicans* and its effect was comparable to amphotericin B. The MIC values of camphor was lower than that of α-pinene against all of the tested microorganisms (Table 2). The MIC and MBC values of camphor for fungi and yeast were 2 μl/ml and were twofold of these values for Gram positive bacteria. The MIC and MBC values of camphor against Gram negative bacteria were 2, 4 μl/ml. α-pinene showed the best antimicrobial activity against *S. aureus*, *B. cereus* and *A. niger*. *C. albicans* was more sensitive than Gram negative bacteria to α-pinene. 1,8-cineole was less effective against all of the tested microorganisms after 24 h. The oil was less active than camphor against all of the tested microorganisms like α-pinene against *A. niger*, *S. aureus*. Oil was more active than 1,8-cineole against Gram positive bacteria, yeast and fungi.

**TABLE 2:** ANTIMICROBIAL ACTIVITY OF ESSENTIAL OIL AND ITS MAIN COMPONENTS BY MICROBROTH DILUTION

Strains	*S. aureus*		*B. cereus*		*E. coli*		*P. aeruginosa*		*C. albicans*		*A. niger*	
						
	MIC	MBC	MIC	MBC	MIC	MBC	MIC	MBC	MIC	MFC	MIC	MFC
1,8-cineole	>8	>8	>8	>8	>8	>8	>8	>8	>8	>8	>8	>8
α- Pinene	4	4	4	8	>8	>8	>8	>8	8	8	4	4
Camphor	1	1	1	1	2	4	2	4	2	2	2	2
Essential oil	8	8	2	2	>8	>8	>8	>8	8	8	8	>8

MIC is minimal inhibitory concentration (μl/ml), MBC is the minimal bactericidal concentration (μl/ml) and MFC is the minimal fungicidal concentration (μl/ml)

In viability assay, log CFU/ml of *S. aureus* for 1,8-cineole decreased more than that for α-pinene and camphor but this value was increased for α-pinene and 1,8-cineole after 60 min. The log CFU/ml of *S. aureus* for camphor decreased continuously. The effect of oil and camphor after 60 min on viable bacteria was increased but log CFU/ml of 1,8-cineole and α-pinene was increased and this effect is decreased (fig. 1). Finally, camphor, α-pinene and 1,8-cineole standards showed antimicrobial activity against all of the tested microorganisms, camphor being the most effective followed by α-pinene and then 1,8-cineole.

**Fig. 1:**
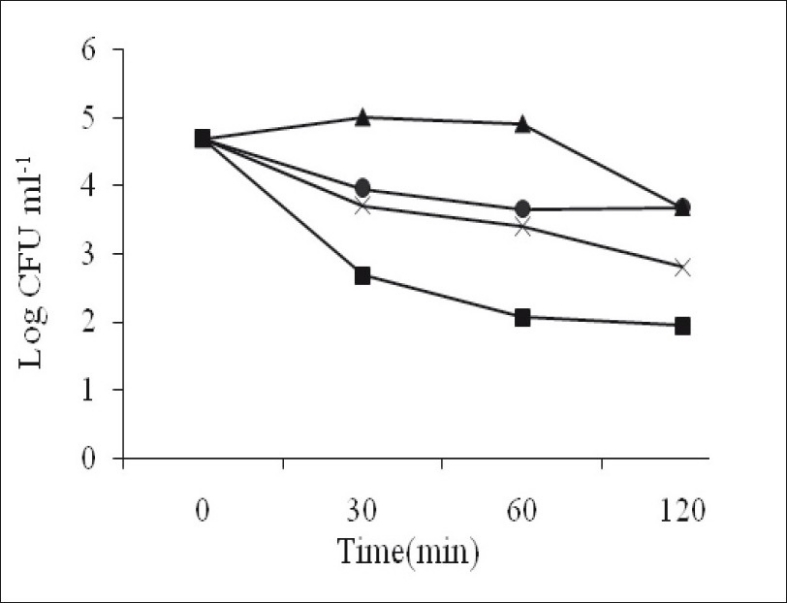
*P. abrotanoides* oil effects and main components on viability of *S. aureus*. Antibacterial effect with time of essential oil of *P. abrotanoides* (–×–) and its main components α-pinene (–●–), 1,8-cineole (–■–) and camphor (–▲–) against *S. aureus*

Plant products have renewed interest in their use as alternative source of antimicrobial compounds[11,12] because problems of the uncontrolled use of synthetic antibiotics[13]. Phytochemicals are small organic biomolecules generally hydrophobic and are designated as naturally occurring antibiotics. Cytoplasmic membrane coagulation, break down of proton motif, breakdown of electron flux and active transport unbalance are some events responsible for providing the antimicrobial property of phytochemicals[14].

*P. abrotanoides* dried root has been successfully used for treatment of cutaneous leishmaniasis in Iranian traditional medicine as poultice[2,3]. In leishmaniasis, the patient may develop a secondary bacterial infection and *S. aureus* is the predominant species[15]. Lesion care and prevent of generation of secondary infections is important in treatment of leishmaniasis. Probably, this plant's leishmanicidal activity has been antimicrobial activity that helps to treat the leishmaniasis.

The Gram negative bacteria were resistant to *P. abrotanoides* oil than Gram positive bacteria. The outer layer of Gram negative bacteria is composed of lipopolysaccharide. This layer forms a hydrophobic permeability barrier which restricts diffusion of hydrophobic compounds through its lipopolysaccharide covering[16]. Essential oils always represent a complex mixture of different chemical components. The nature and proportion of the individual constituents of the oil could influence on their antimicrobial activity. 1,8-cineole is a lipophilic compound and the lipophilic property of α-pinene is more than camphor. Generally, a more lipophilic compound had greater affinity for cell membranes and had greater toxicity. Therefore, 1,8-cineole had the best effect on viability of *S. aureus* and the effect of α-pinene and then camphor was lower. After 24 h, the effect of camphor on viability of bacteria is more than that of α-pinene and 1,8-cineole. Furthermore, the lipophilic structure of components, the difference in antimicrobial activity of components is attributed to aqueous solubility of components. Uptake of monoterpenes will be determined by both its aqueous solubility and the permeability of the outer envelope of microorganisms[10]. The aqueous solubility of camphor is larger than others and had more antimicrobial activity than other components against all of the microorganisms. 1,8-cineole are inactive probably because was unable to effectively penetrate the outer membrane for long time. Camphor and α-pinene were found in high concentration in *P. abrotanoides* oil and possess antimicrobial activity. High concentrations of α-pinene and camphor in the essential oil are probably an explanation for the antimicrobial activity possessed by *P. abrotanoides* against Gram positive bacteria and yeast. α-Pinene destroys the cellular integrity of Gram positive bacteria and inhibit respiratory activity in yeast mitochondria and had some antifungal activity but Gram negative bacteria were more resistant to it[17].

It was shown that 1,8-cineole was inactive against Gram positive bacteria[18] but other study showed 1,8-cineole had some antifungal activity[19] and high insecticidal property[20]. The MIC values of oil were usually lower than those of their constituents. One reason for this result could be synergistic action of components. Therefore, the antibacterial activity of oil from *P. abrotanoides* can be attributed to its major components and/or trace compounds and possible synergistic and antagonistic effect of compounds in the oil. *S. aureus* is most sensitive organism to *P. abrotanoides* oil. *S. aureus* is an important secondary pathogenic bacterium in leishmaniasis which may play an important role in the size and shape of the lesion, as well as in scar development[5]. Treatment of secondary infection is required and elimination of parasites after treatment was followed. Therefore, *P. abrotanoides* can be used as antibacterial and antileishmanial agent for treatment of leishmaniasis. Some *in vivo* clinical studies should be done for evaluating of this effect.
